# DNA methylation of the *PLIN1* promoter downregulates expression in chicken lines

**DOI:** 10.5194/aab-62-375-2019

**Published:** 2019-07-03

**Authors:** Yuhang Sun, Rui Li, Guiying Zhai, Xinyang Zhang, Yuxiang Wang

**Affiliations:** 1 Key Laboratory of Chicken Genetics and Breeding, Ministry of Agriculture and Rural Affairs, Northeast Agricultural University, Harbin 150030, China; 2 Key Laboratory of Animal Genetics, Breeding and Reproduction, Education Department of Heilongjiang Province, Heilongjiang 150030, China; 3 College of Animal Science and Technology, Northeast Agricultural University, Harbin, Heilongjiang 150030, China

## Abstract

Evidence suggests that Perilipin-1 (*PLIN1*) is subject to functional regulation by epigenetic modifications in women with obesity. However, whether chicken *PLIN1* expression is regulated by DNA methylation is unknown. Here, Sequenom MassARRAY and real-time polymerase chain reaction (PCR) were conducted to analyze the promoter methylation status and expression of the *PLIN1* gene in Northeast Agricultural University broiler lines divergently selected for abdominal fat content. We found that chicken *PLIN1* expression was significantly higher in adipose tissue of fat-line broilers than in lean lines at 1–7 weeks of age, and was significantly positively correlated with abdominal fat percentage (AFP) in chicken adipose development (Pearson's r=0.627, P<0.001). The region analyzed for DNA methylation was from -12 to -520 bp upstream of the translation start codon ATG, and had five CpG sites, where only the DNA methylation levels of CpG5 located at position -490 bp were significantly higher in lean compared to fat chickens at 5 and 6 weeks (P<0.05) and were significantly negatively correlated with *PLIN1* mRNA levels and AFP (P<0.05). These results shed new light on the regulation of hypertrophic growth in chicken adipose development.

## 1 Introduction

Obesity is characterized by an expansion of white adipose tissue (WAT) mass resulting from increased adipocyte number and/or size. One of the most important components of mature adipocytes is lipid droplets with intracellular space almost occupied by lipid droplets. The degree of adipocyte differentiation mainly depends on the size of lipid droplets. Perilipin-1 (*PLIN1*), a lipid-droplet-associated protein, was originally identified in adipocytes (Greenberg et al., 1991). Our group showed that lipid droplets are surrounded by *PLIN1* in chicken adipocytes at different time points after cell differentiation (Qin et al., 2016). In the basic condition, overexpression of *PLIN1* promotes chicken preadipocyte lipid accumulation (Zhou et al., 2012). With hormone stimulation, overexpression of *PLIN1* inhibits lipid accumulation in adipocyte cells, consistent with findings in mammals (Miyoshi et al., 2008, 2007, 2006).

Obesity is associated with increased basal lipolysis and decreased levels of *PLIN1* protein in adipose tissue (Mottagui-Tabar et al., 2003; Ray et al., 2009; Wang et al., 2003). Evidence suggests that *PLIN1* is subject to functional regulation by epigenetic modifications in women with obesity (Bialesova et al., 2017). However, the DNA methylation status of the *PLIN1* gene and its role in chicken adipose development has not been elucidated. To enhance our understanding of molecular mechanisms underlying chicken adipose tissue development and adipogenesis, investigating the DNA methylation status of *PLIN1* and its effect on chicken adipose development is essential.

Northeast Agricultural University broiler lines divergently selected for abdominal fat content (NEAUHLF) have been selected by long-term divergent selection since 1996 using abdominal fat percentage (AFP) and plasma very low-density lipoprotein (VLDL) concentration (Liu et al., 2007). After 19 generations of selection, differences in AFP and abdominal fat weight (AFW) were striking. *PLIN1* is a critical regulator of fat storage and breakdown and its expression is significantly higher in adipose tissue of fat-line broilers than lean broilers at 7 weeks (Wang et al., 2011). This result suggests a significant association between *PLIN1* expression and abdominal fat content. DNA methylation is important for regulation of gene expression and is critical in establishing patterns of gene repression during development (Cedar and Bergman, 2009). We hypothesized that the chicken *PLIN1* gene is differently methylated in the lean and fat chicken lines during adipose tissue development.

Here, we used Sequenom MassARRAY and real-time PCR to analyze the promoter methylation status and expression of the *PLIN1* gene in abdominal adipose of lean and fat chicken lines at 1–7 weeks of age. Our findings showed a positive correlation between AFP and *PLIN1* mRNA levels in chicken adipose development, and DNA methylation levels of CpG5 were significantly higher in lean compared to fat chickens at 5 and 6 weeks and were significantly negatively correlated with *PLIN1* mRNA levels and AFP. This result suggests that epigenetic regulation of *PLIN1* might be important for hypertrophic growth in chicken adipose development.

## 2 Materials and methods

### 2.1 Animals and tissues

Animal work was carried out in accordance with the guidelines for the care and use of experimental animals established by the Ministry of Science and Technology of the People's Republic of China (approval number: 2006-398) and the Laboratory Animal Management Committee of Northeast Agricultural University (Harbin, P. R. China). Chickens from NEAUHLF generation 19 (G19) were used. All birds were housed under similar environmental conditions with free access to feed and water. The abdominal fat tissues, abdominal fat pad and adipose tissue surrounding the gizzard from each individual male bird were stripped and weighed as AFW, then snap-frozen in liquid nitrogen and stored at -80${}^{\circ }$ for extraction of genomic DNA and total RNA. A total of 70 male birds (five birds per line per time point) at 1–7 weeks of age were used in this process. AFP = AFW/body weight.

### 2.2 RNA isolation and quantitative real-time RT-PCR

Total RNA from abdominal adipose tissues was extracted by using TRIzol (Invitrogen, Carlsbad, CA, USA) following the manufacturer's protocol and treated with DNase I (Takara, Dalian, China), via visualization of the 18S and 28S ribosomal RNA bands on a denaturing formaldehyde agarose gel to assess RNA quality. Complementary DNAs were synthesized in a final volume of 20 µL with 1 mg of total RNA, an oligo(dT) anchor primer and ImProm-II reverse transcriptase (Promega, Madison, WI, USA). Conditions were 25 ${}^{\circ }$C for 5 min, 42 ${}^{\circ }$C for 60 min and 70 ${}^{\circ }$C for 15 min.

Quantitative relative-transcription PCR (RT-PCR) was performed with the 7500 real-time PCR system (Applied Biosystems, Foster City, CA, USA) using FastStart Universal SYBR Green Master kits (Roche, Shanghai, China). From each RT reaction, within a 10 µL reaction add 1 µL of product; reaction mixtures were incubated in an ABI Prism 7500 sequence detection system (Applied Biosystems) at one cycle at 95 ${}^{\circ }$C for 10 min, 40 cycles of 95 ${}^{\circ }$C for 15 s and 60 ${}^{\circ }$C for 1 min. To detect and eliminate possible primer-dimer artifacts, we using Dissociation Curve 1.0 software (Applied Biosystems) to analyze the dissociation curves for each PCR reaction. The relative expression of *PLIN1* was calculated using the formula 2${}^{\Delta -\mathrm{CT}}$ with TATA-box binding protein (*TBP*) as an internal reference. Primers used for quantitative RT-PCR were shown in Table 1.

**Table 1:** Primers used.

Orientation	Sequence (5${}^{\prime }$-3${}^{\prime }$)	$T_{m}$
		valve (${}^{\circ }$)
Sequenom MassARRAY-*PLIN1* (NM_001127439)		
Forward	aggaagagagTGTGGTGTTGGGGTATTATTATATTT	60
Reverse	cagtaatacgactcactatagggagaaggctTAAATAACCTAACCTTTTCCTCCCA	
RT-PCR SYBR Green-*PLIN1* (NM_001127439)		
Forward	GCCAAGGAGAACGTGCT	60
Reverse	TCACTCCCTGCTCATAGACC	
RT-PCR SYBR Green-TBP (NM_205103)		
Forward	GCGTTTTGCTGCTGTTATTATGAG	60
Reverse	TCCTTGCTGCCAGTCTGGAC	

$T_{m}$: melting temperature.

### 2.3 Methylation analysis

The Sequenom MassARRAY platform was used to determine methylation levels of loci in the CpG island promoter of selected *PLIN1* genes (GeneBank accession no. GU327532.1). CpG islands were predicted using CpG Island Searcher software (https://www.ebi.ac.uk/Tools/seqstats/emboss_cpgplot/, last access: 3 June 2019). Thresholds were GC > 50 %, CpG observed / expected value >0.6 and CpG island length >200 bp.

Adipose tissue DNA was extracted and isolated with QIAamp DNA Mini Kit (Qiagen, Hilden, Germany) following the manufacturer's protocol. NanoDrop spectrophotometer from GE Healthcare Life Science (Uppsala, Sweden) was conducted to quantify the genomic DNA. Bisulfite conversation of the DNA was performed using the EpiTect Bisulfite Kit (Qiagen, Hilden, Germany) following the manufacturer's protocol. The online software EpiDesigner (https://www.epidesigner.com,last access: 3 June 2019, Agena Bioscience, USA) was used to design PCR primers, and primer sequences were shown in Table 1. PCR products were used as a template for transcription and base-specific cleavage reactions using the MassCLEAVE kit (Sequenom, USA). DNA methylation levels of fragmented samples were quantified by a MassARRAY analyzer compact matrix-assisted laser desorption/ionization time-of-flight mass spectrometry instrument and EpiTYPER analyzer software (Sequenom). Individual CpG sites or clusters of consecutive CpG sites were defined as CpG units following the manufacturer's protocol.

### 2.4 Statistical analysis

All data are presented as means plus standard error (SE). Differences between groups were analyzed using unpaired, two-tailed Student's t tests. Pearson's r was used to analyze the correlation between AFP and mRNA levels, methylation and mRNA levels, and AFP and methylation. P<0.05 was considered significant.

**Figure 1:**
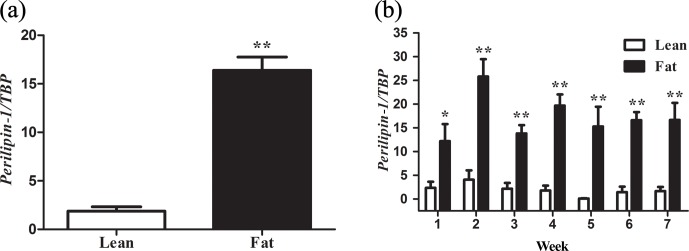
*PLIN1* expression in NEAUHLF abdominal adipose tissues. The mRNA levels were determined by real-time quantitative reverse-transcription polymerase chain reaction and normalized to TATA-box binding protein (*TBP*) mRNA measured in parallel experiments. Results are the mean ± SE. **(a)** Mean *PLIN1* expression in adipose tissues of lean and fat broilers (n=35). **(b)** Mean *PLIN1* expression in adipose tissues of lean and fat broilers at 1–7 weeks of age (n=5). ${}^{*}\;P<0.05$ and ${}^{**}\;P<0.01$.

**Table 2:** AFW and AFP in lean and fat lines of NEAUHLF.

		Mean AFW and AFP of each week (n=5)						
Traits	Line	1 week	2 weeks	3 weeks	4 weeks	5 weeks	6 weeks	7 weeks
AFW	Lean	$0.22\pm 0.04^{*}$	$1.21\pm 0.15^{*}$	$2.15\pm 0.29^{*}$	$2.35\pm 0.21^{*}$	$2.68\pm 0.32^{*}$	$6.43\pm 0.43^{*}$	$11.87\pm 1.91^{*}$
(g)	Fat	$0.92\pm 0.15^{*}$	$4.22\pm 0.69^{*}$	$13.55\pm 1.28^{*}$	$19.45\pm 1.98^{*}$	$33.62\pm 2.96^{*}$	$53.02\pm 6.30^{*}$	$86.56\pm 3.68^{*}$
AFP	Lean	$0.18\pm 0.03^{*}$	$0.52\pm 0.06^{*}$	$0.55\pm 0.07^{*}$	$0.41\pm 0.03^{*}$	$0.36\pm 0.04^{*}$	$0.52\pm 0.04^{*}$	$0.67\pm 0.07^{*}$
(%)	Fat	$0.78\pm 0.13^{*}$	$1.83\pm 0.30^{*}$	$3.41\pm 0.30^{*}$	$3.32\pm 0.26^{*}$	$4.19\pm 0.33^{*}$	$3.95\pm 0.28^{*}$	$4.94\pm 0.18^{*}$

Comparison of lean and fat lines using unpaired Student's t test. Values are the mean ± SE. ${}^{*}$ Significant difference in AFW and AFP between the two chicken lines (P<0.01).

**Table 3:** Correlations between the average methylation level of promoter region and *PLIN1* expression level (n=10).

Week	Pearson's r	P value
1 week	-0.1373	0.7053
2 weeks	-0.7242	0.0179
3 weeks	0.3496	0.3221
4 weeks	0.5251	0.1191
5 weeks	-0.3421	0.3333
6 weeks	-0.5235	0.1205
7 weeks	-0.1442	0.6910

## 3 Results

### 3.1 *PLIN1* and abdominal fat deposition

We used 70 male birds that had normal weight in all test weeks of age (Fig. S1 in the Supplement). AFP and AFW were calculated in G19 (Table 2). AFW and AFP were significantly different between the two lines in adipose development and AFP in the fat line at 7 weeks of age was 7.37 times higher than the lean line (Table 2). In our previous study, in G11, *PLIN1* expression was significantly higher in adipose tissue of fat-line broilers than lean broilers at 7 weeks (Wang et al., 2011). To study if *PLIN1* expression was related to AFP, the relationship between *PLIN1* mRNA levels in abdominal fat tissue and abdominal fat content was analyzed. *PLIN1* transcript levels in G19 fat males were higher than in lean males at 1–7 weeks of age (Fig. 1) (P<0.05). With increased age, no significant difference was seen in *PLIN1* expression, which maintained a relatively low level in the lean line (Fig. 1b). Correlation analysis showed a significant positive correlation between AFP and *PLIN1* mRNA levels in chicken adipose development (Pearson's r=0.627, P<0.001).

**Figure 2:**
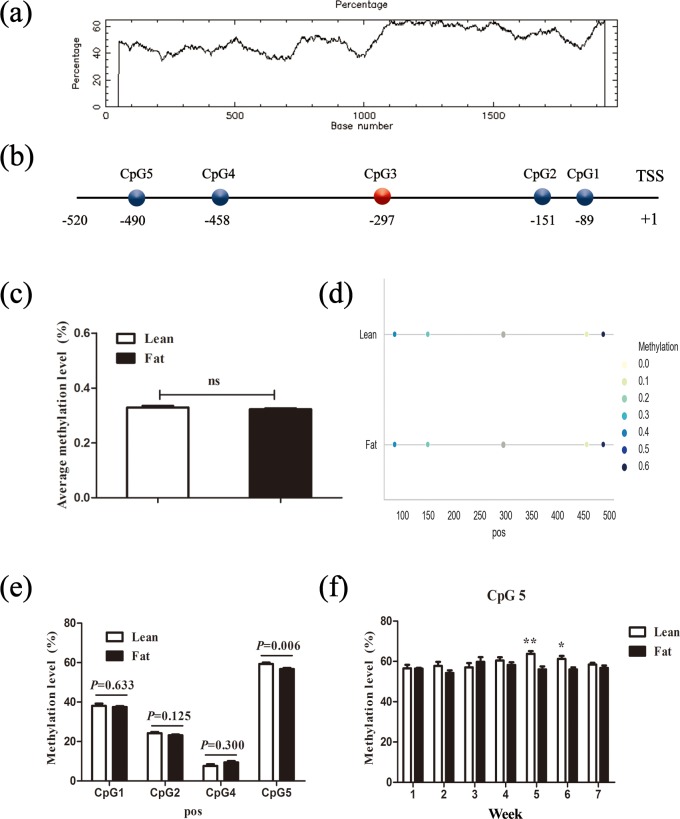
Comparison of CpG site methylation at the chicken *PLIN1* promoter in NEAUHLF. **(a)** Search for CpG islands in the chicken *PLIN1* promoter. Thresholds were GC >50 %, CpG observed/expected value >0.6 and CpG island length >200 bp. **(b)** Schematic diagram of *PLIN1* gene promoter. All numbered positions are relative to the adenine of the translation start site of chicken *PLIN1*. Five CpG sites were found in the analyzed region, in which CpG3 (red) was not detected. **(c)** Average methylation level of promoter region in line and fat lines. Panels **(d)** and **(e)** show average methylation levels of the *PLIN1* promoter CpG sites in adipose tissues of lean and fat broilers in all weeks (n=35). **(f)** Average methylation levels of CpG5 in adipose tissues of lean and fat broilers at 1–7 weeks of age (n=5). ${}^{*}\;P<0.05$ and ${}^{**}\;P<0.01$.

### 3.2 DNA methylation of chicken *PLIN1* promoter in adipose tissue

To determine whether the *PLIN1* gene expressed in chicken abdominal adipose tissue is regulated by DNA methylation, we used CpG Searcher software. Bioinformatics analysis revealed that the 2 kb promoter region of chicken *PLIN1* had no typical CpG islands (Fig. 2a). Then, we investigated the methylation status within a 509 bp region from -520 to -12 bp upstream of the translation start codon ATG that contained *PLIN1* core promoter regions (Zhou et al., 2016). Five CpG sites were found in the analyzed region, at positions -89, -151, -297, -458 and -490 bp (Fig. 2b). The sites were named CpG1 through CpG5. We quantified the methylation levels of CpG units within the promoter using Sequenom MassARRAY technology. For technical reasons, the CpG3 site (red, Fig. 2b) could not be detected. In summary, the average methylation level of the promoter region, with five CpG sites, was not significantly different in the lean line compared to the fat line (Fig. 2c). Then, we tested the relationship between the average methylation level of promoter region and *PLIN1* expression level at all tested ages, and only the promoter methylation at 2 weeks of age showed a significantly negative correlation with *PLIN1* expression (Pearson's r=-0.724, P=0.018) (Table 3).

Single CpG site methylation analysis showed that hypermethylated CpG sites were not found in the two lines in the *PLIN1* promoter region, and only average DNA methylation levels of CpG5 at position -490 bp were significantly higher in lean than fat chickens (P=0.006) (Fig. 2d, e). Further analysis showed that CpG5-site methylation levels were significantly higher in the lean line than in the fat line at 5 and 6 weeks (P<0.05, Fig. 2f). Then, we tested correlations between average DNA methylation level of each CpG site and *PLIN1* expression level. The results showed that, of all tested CpGs, only the average methylation level of CpG5 at -490 bp displayed a significantly negative correlation with *PLIN1* expression level (Pearson's r=-0.319, P=0.007) (Table 4), and was negatively correlated with AFP at 5 and 6 weeks of age (Pearson's r=-0.894, P<0.001 and Pearson's r=-0.637, P=0.047, respectively).

## 4 Discussion

Studies suggest that *PLIN1* is highly expressed in white adipocytes and is actively involved in lipolysis regulation through interaction with hormone-sensitive lipase and lipase activator CGI-58 (adipose triglyceride lipase) (Contreras et al., 2017; Granneman et al., 2009). *PLIN1* knockout increases basal lipolysis and decreases liquid droplet (LD) size in adipocytes and causes resistance to diet-induced obesity in mice (Martinez-Botas et al., 2000; Tansey et al., 2001). *PLIN1*${}^{-/-}$ mice are lean, with normal body weight but reduced WAT stores. Furthermore, *PLIN1*${}^{-/-}$ mice are resistant to diet and genetically induced obesity (Castro-Chavez et al., 2003; Greenberg et al., 1991; Martinez-Botas et al., 2000; Saha et al., 2004; Tansey et al., 2001).

Our results indicated that *PLIN1* expression was significantly higher in fat lines than in lean lines at all tested ages. Our results also demonstrated a significant positive correlation between AFP and mRNA expression in chicken adipose development. This result indicated that *PLIN1* might be a marker gene for selection for fatness.

Adipocyte gene transcription is modulated by epigenetic mechanisms. Chicken PPARγ is regulated by DNA methylation during adipose tissue development (Sun et al., 2014) and DNA methylation may regulate CEBPA expression in early chicken adipose development (Gao et al., 2015). Dysregulated CpG methylation of lipolysis genes is a major feature of the adipocyte epigenetic signature in women with obesity and epigenetic regulation of *PLIN1* is important for increased adipocyte lipolysis in insulin-resistant states such as obesity (Bialesova et al., 2017). In this study, we did not find a CpG island in the upstream 2.0 kb of the translation start codon of chicken *PLIN1*. Only in four CpG loci detected in the core promoter region of the gene, did we find that CpG5 was negatively correlated with chicken *PLIN1* mRNA expression, and the DNA methylation level of this locus was negatively correlated with AFP in broilers at 5 and 6 weeks of age. Studies on the development of adipose tissue in chickens show that increases in the abdominal fat pad mass of broiler chickens mainly depends on hyperplasia of adipocytes until 4 weeks of age and hypertrophic growth beyond 4 weeks (Hood, 1982). *PLIN1* can augment triglyceride synthesis and promote enlargement of lipid droplets (LDs), leading to the formation of large LDs (Koltes and Spurlock, 2011; Sun et al., 2013). Therefore, we speculate that DNA methylation can negatively regulate the expression of the *PLIN1* gene during the growth and development of adipose tissue in broiler chickens, affect the hypertrophy of adipocytes and then inhibit the accumulation of body fat. In addition, we also found that adding methyltransferase inhibitors significantly increased the expression of the *PLIN1* gene in DF-1 cells (Date not listed). In short, the influences of promoter DNA methylation on *PLIN1* gene expression shed new light on the regulation of hypertrophic growth in chicken adipose development.

**Table 4:** Correlations between each DNA methylation site and *PLIN1* expression.

	CpG sites (n=70)			
	-87 bp	-151 bp	-458 bp	-490 bp
Pearson's r	-0.067	-0.110	0.128	-0.319
P value	0.581	0.367	0.302	0.007

Future work is needed to define how CpG methylation interacts with other known regulators of *PLIN1* mRNA expression. Mammalian studies show that DNA methylation, including hypermethylation and hypomethylation, are important for regulating the expression of transcription factors, transcriptional cofactors and other genes involved in mammalian adipose development and adipogenesis (Bowers et al., 2006; Noer et al., 2007; Shore et al., 2010). CpG methylation at promoter regions has been widely recognized as an effective epigenetic modification, which prohibits transcription factor recruitment, resulting in transcription suppression (Hu et al., 2013). We used TFBIND (http://tfbind.hgc.jp/, last access: 3 June 2019) and JASPAR (http://jaspar.genereg.net/, last access: 3 June 2019) and to predict transcription factor binding sites around the promoter region detected in this study. TFBIND results showed that the CpG5 site match reported binding motifs for NF-κB and E2F family members. NF-κB is the well-known transcription factor regulating *PLIN1* expression (Laurencikiene et al., 2007). Recent evidence suggests that a cytosine at the -1 position of a κB site (-1C) could be methylated, which thereafter impaired NF-κB binding and/or function (Wang et al., 2017). However, it is unknown whether the methylation of CpG5 will affect NF-κB's binding to its binding site. CpG methylation differentially regulates the response of certain E2F elements to different E2F family members (Campanero et al., 2000). The E2F consensus motif contained only one methylation CpG and did not affect binding of E2F2-5, but abrogated E2F1's binding (Campanero et al., 2000). Therefore, the CpG5 site may be an E2F1 binding site and methylation of CpG might influence E2F1's function. However, to date, there is no report of E2F1 regulating *PLIN1* expression.

In addition, multiple binding motifs for transcription factors, such as C/EBPβ, NRF1, KLF4 and KLF5, were found around the CpG5 site (±30 bp). Evidence suggests that KLF4 can activate *C/EBP*β expression, and C/EBPβ and KLF5 can co-activate *PPAR*γ expression and promote adipocyte differentiation (Oishi et al., 2005; Birsoy et al., 2008). NRF1 has been implicated in lipid droplet accumulation, negative regulation of the P1 promoter of *PPAR*γ gene, and inhibition of chicken adipogenesis (Cui et al., 2018; Liu et al., 2008). Although those transcription factors have effects on adipocyte differentiation, CpG5 prevents or promotes which transcription factors' bind to influence the expression regulating of *PLIN1* gene. Further research is needed.

Taken together, in this study, we suggested that the *PLIN1* gene was a marker gene for selection of fat traits. The DNA methylation of CpG5 at position -490 bp of the *PLIN1* promoter has a certain impact on *PLIN1* gene expression. Our results imply that epigenetic regulation of *PLIN1* might be important for hypertrophic growth in chicken adipose development.

## Supplement

10.5194/aab-62-375-2019-supplement The supplement related to this article is available online at: https://doi.org/10.5194/aab-62-375-2019-supplement.
