# Supplementary material for: DNA methylation of the PLIN1 promoter downregulates expression in chicken lines
Source: Arch Anim Breed. 2019 Jul 3;62(2):375–82. doi: 10.5194/aab-62-375-2019 (PMC6852845; doi:10.5194/aab-62-375-2019)
Supplement: The supplement related to this article is available online at: https://doi.org/10.5194/aab-62-375-2019-supplement. [file aab-62-375-supplement.pdf]

Supplement of Arch. Anim. Breed., 62, 375–382, 2019
https://doi.org/10.5194/aab-62-375-2019-supplement
© Author(s) 2019. This work is distributed under
the Creative Commons Attribution 4.0 License.

Open Access
Archives Animal Breeding

*Supplement of*

# DNA methylation of the *PLIN1* promoter downregulates expression in chicken lines

**Yuhang Sun et al.**

*Correspondence to:* Yuxiang Wang (wyx2000@neau.edu.cn)

The copyright of individual parts of the supplement might differ from the CC BY 4.0 License.

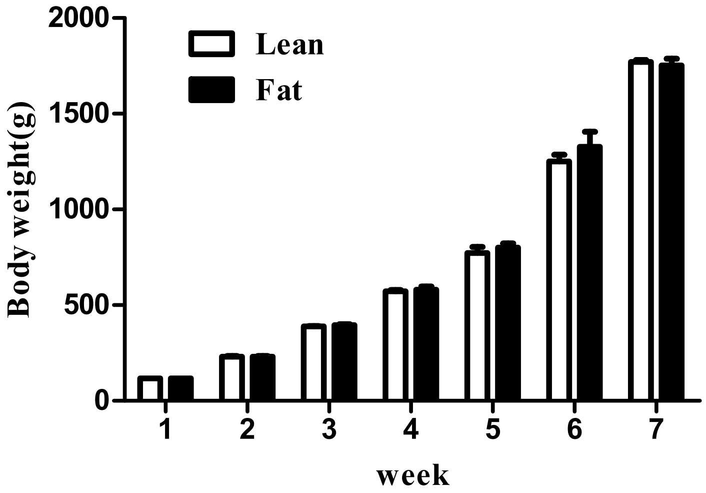

Lean
Fat
Body weight(g)
2000
1500
1000
500
0
1
2
3
4
5
6
7
week
